# Prefused lysosomes cluster on autophagosomes regulated by VAMP8

**DOI:** 10.1038/s41419-021-04243-0

**Published:** 2021-10-13

**Authors:** Qixin Chen, Mingang Hao, Lei Wang, Linsen Li, Yang Chen, Xintian Shao, Zhiqi Tian, Richard A. Pfuetzner, Qing Zhong, Axel T. Brunger, Jun-Lin Guan, Jiajie Diao

**Affiliations:** 1 Department of Cancer Biology, University of Cincinnati College of Medicine, Cincinnati, OH 45267 USA; 2 State Key Lab of Animal Nutrition, China Agricultural University, Beijing, 100193 China; 3 Department of Molecular and Cellular Physiology, Stanford University, Stanford, 94305 CA USA; 4 Howard Hughes Medical Institute, Stanford University, Stanford, 94305 CA USA; 5 Key Laboratory of Cell Differentiation and Apoptosis of Chinese Ministry of Education, Department of Pathophysiology, Shanghai Jiao Tong University School of Medicine (SJTU-SM), Shanghai, 200025 China

**Keywords:** Cancer therapeutic resistance, Membrane fusion

## Abstract

Lysosome–autophagosome fusion is critical to autophagosome maturation. Although several proteins that regulate this fusion process have been identified, the prefusion architecture and its regulation remain unclear. Herein, we show that upon stimulation, multiple lysosomes form clusters around individual autophagosomes, setting the stage for membrane fusion. The soluble N-ethylmaleimide-sensitive factor attachment protein receptor (SNARE) protein on lysosomes—vesicle-associated membrane protein 8 (VAMP8)—plays an important role in forming this prefusion state of lysosomal clusters. To study the potential role of phosphorylation on spontaneous fusion, we investigated the effect of phosphorylation of C-terminal residues of VAMP8. Using a phosphorylation mimic, we observed a decrease of fusion in an ensemble lipid mixing assay and an increase of unfused lysosomes associated with autophagosomes. These results suggest that phosphorylation not only reduces spontaneous fusion for minimizing autophagic flux under normal conditions, but also preassembles multiple lysosomes to increase the fusion probability for resuming autophagy upon stimulation. VAMP8 phosphorylation may thus play an important role in chemotherapy drug resistance by influencing autophagosome maturation.

## Introduction

Lysosomes are acidic organelles ranging from 0.025 to 0.8 μm in diameter that are the degradation centers of cellular materials in eukaryotic cells [1, 2]. Because lysosomal dysfunction is associated with lysosomal storage disorders, aging, and cancer, an understanding of lysosomal dynamics can aid investigations into the pathogenesis of lysosome-related diseases [3, 4]. Lysosomes are involved in various metabolic processes at the cellular level, including autophagy, by which cells recycle their contents for survival [5, 6]. In that process, the fusion of lysosomal and autophagosomal membranes is crucial for autophagosome maturation to occur. Although much attention has been paid to protein factors that regulate their fusion, the role of lysosomal dynamics in the process remains unknown. Interestingly, grouped lysosomal movements have been observed [7], but the underlying mechanism is unknown.

Organelles not only play unique roles, but they also interact with each other in organelle interaction networks to rapidly exchange materials and communicate while participating in various cellular processes [8, 9]. For example, fragmented mitochondria undergo a process of assembly upon interacting with F-actin during their clearance [10], and ribosomes regulate the biophysical properties of the cytoplasm by crowding [11]. Although such observations on the important role of organelle dynamics imply biological functions of lysosome clustering, the current understanding of the details of such clustering has been hindered by the resolution limits of conventional microscopes [12]. Super-resolution fluorescence microscopy methods—stimulated emission depletion [13], structured illumination microscopy (SIM) [14], stochastic optical reconstruction microscopy [15], and photo-activated localization microscopy [16]—now enable investigation of dynamic interaction among organelles at the subcellular level in living cells [12].

The prefusion architecture involving fusion proteins and membranes determines the fusion pathway. For instance, a prefusion point contact between two membranes can directly lead to full fusion upon triggering [17], and protein-induced membrane curvature may accelerate fusion [18, 19]. Meanwhile, the proper assembly of the prefusion state involving several proteins can significantly reduce the energy barrier to the fusion of two membranes and set the stage for regulation of the fusion process, for example by Ca^2+^-triggering [20]. However, for the fusion of lysosomal and autophagosomal membranes mediated by autophagic soluble *N*-ethylmaleimide-sensitive factor attachment protein receptors (SNAREs), neither the architecture of the prefusion state nor regulatory mechanisms have been identified.

In this study, we employed SIM to monitor the dynamics of the clustering of lysosomes upon stimulation. We observed a prefusion state of clustered lysosomes associated with autophagosomes that could supply more membrane contacts and in turn, improve the efficiency of fusion. We also found that the vesicle-associated membrane protein 8 (VAMP8), the SNARE protein on lysosomes, regulates the lysosome–autophagosome association. Finally, phosphorylation of VAMP8 reduces fusion by preventing full SNARE complex assembly, suggesting a new regulatory mechanism for cell fate.

## Results

### Super-resolution imaging allows tracking the formation of lysosome clusters upon stimulation

**Fig. 1:**
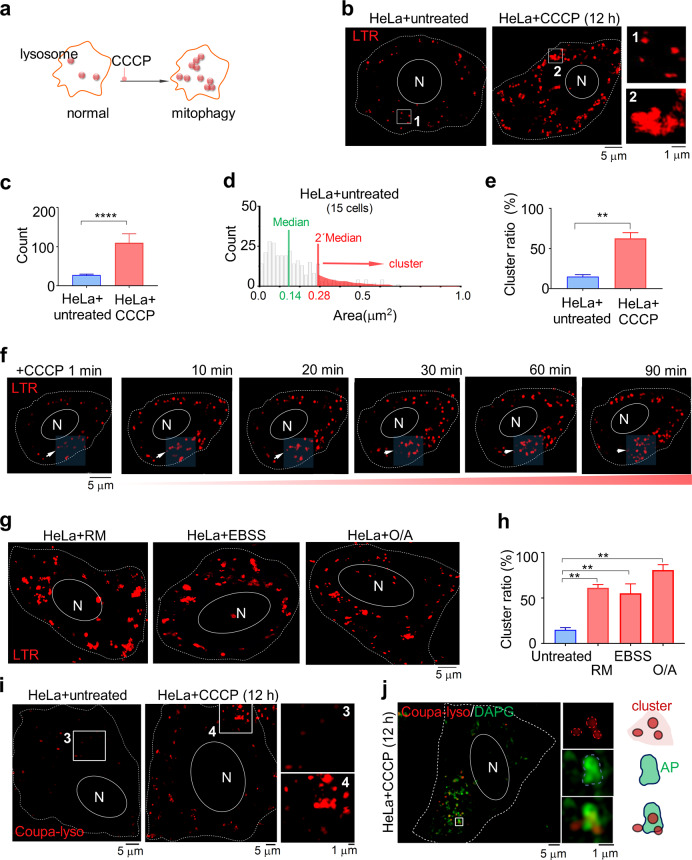
Super-resolution imaging of lysosome clustering upon stimulation. **a** Schematic representation of the formation of lysosome clusters in CCCP-induced mitophagy. **b** Formation of lysosome clusters in HeLa cells with or without CCCP-treated for 12 h. Lysosomes were stained by 200 nM commercial LysoTracker Red (LTR) at 37 °C for 30 min and then observed using SIM. White rectangles 1 and 2 show an enlarged area; white dotted lines indicate cell membranes; N, nucleus. **c** Lysosome puncta count in HeLa cells with and without CCCP treatment. **d** Distributions of lysosome areas in untreated HeLa cells. **e** Ratio of clustered lysosome clusters to the total number of lysosomes in untreated and CCCP-treated HeLa cells. **f** SIM revealing the dynamic process of the formation of lysosome clusters in CCCP-treated HeLa cells. The white arrow shows a representative dynamic formation of a lysosome cluster. The lower right number indicates the ratio of lysosome clusters to lysosomes. **g** SIM images and (**h**) qualification of the formation of lysosome clusters in RM-, EBSS-, and O/A-treated HeLa cells. **i** Specific lysosome probe, Coupa-lyso, was used for revealing lysosome cluster in living cells. Cells were stained with Coupa-lyso without (left) or with (right) CCCP treatment. White rectangles indicate the enlarged region. **j** CCCP-treated cells co-stained with Coupa-lyso and DAPG, showing lysosome cluster contact with autophagosome. White rectangles indicate representative clustered lysosomes around autophagosome. Data in **c**, **e** and **h** are presented as mean ± SEM, *p < 0.05, **p < 0.01, ***p < 0.001, ****p < 0.0001.

The limited resolution of conventional microscopy has hindered investigations of lysosome interactions with other organelles at the nanoscale level. To overcome this limitation and examine lysosomal dynamics under physiological and pathological conditions, we previously employed SIM, a super-resolution fluorescence imaging technique for living cells, to study mitochondria-lysosome interactions [21, 22]. To study lysosomal dynamics in autophagy, we used SIM to image lysosomes upon inducing autophagy by several different agents. First, HeLa cells were treated with 10 μM carbonyl cyanide *m*-chlorophenylhydrazone (CCCP) [23–25], a common inducer of mitophagy, for 12 h before using staining to monitor lysosome cluster under SIM (Fig. 1a). Compared to untreated HeLa cells, a substantial level of lysosome clustering was visible (Fig. 1b), along with an increased number of lysosome puncta (Fig. 1c). To quantify lysosome clusters, we used SIM to measure the size distribution of lysosome spots (Fig. 1d). In that process, we define a *lysosome cluster* as a cross-sectional area of lysosome puncta larger than twice the median area of lysosomes in SIM-captured images (Fig. 1d). We found that CCCP treatment significantly enhanced the formation of lysosome clusters (Fig. 1e). Moreover, we observed a dynamic process in which lysosomes were converted from a freely diffusing state to a clustered state (Fig. 1f). The formation of lysosome clusters was also found in other cell lines, including A549, PC12, and SLC-80 (Supplementary Fig. S1a).

We also used other inducers of autophagy: rapamycin (RM) [26, 27], Earle’s balanced salt solution starvation medium (EBSS) [28], and the ATP synthesis inhibitor oligomycin A (O/A) [29], to compare their effects on the formation of lysosome clusters (Fig. 1g). As with CCCP treatment, lysosome count (Supplementary Fig. S2) and lysosome clustering (Fig. 1h) significantly increased in all of these stimulations. Subsequently, we used confocal microscopy to monitor lysosome clustering and detected large lysosome spots in RM-treated, EBSS-treated, and O/A-treated HeLa cells (Supplementary Fig. S1b). Our results suggest that lysosomes form clusters upon induction of autophagy.

LysoTracker Red (LTR) is known to stain autolysosomes as well. We then used another lysosome specific tracker, Coupa-lyso, which goes to a dark state upon the formation of autolysosomes in mitophagy [25]. As shown in Fig. 1i, lysosome clustering was also observed with Coupa-lyso. Moreover, together with another autophagosome dye, DAPG, we found lysosome clustering may form around autophagosome (Fig. 1j).

### Lysosome clusters associate with autophagosomes

**Fig. 2:**
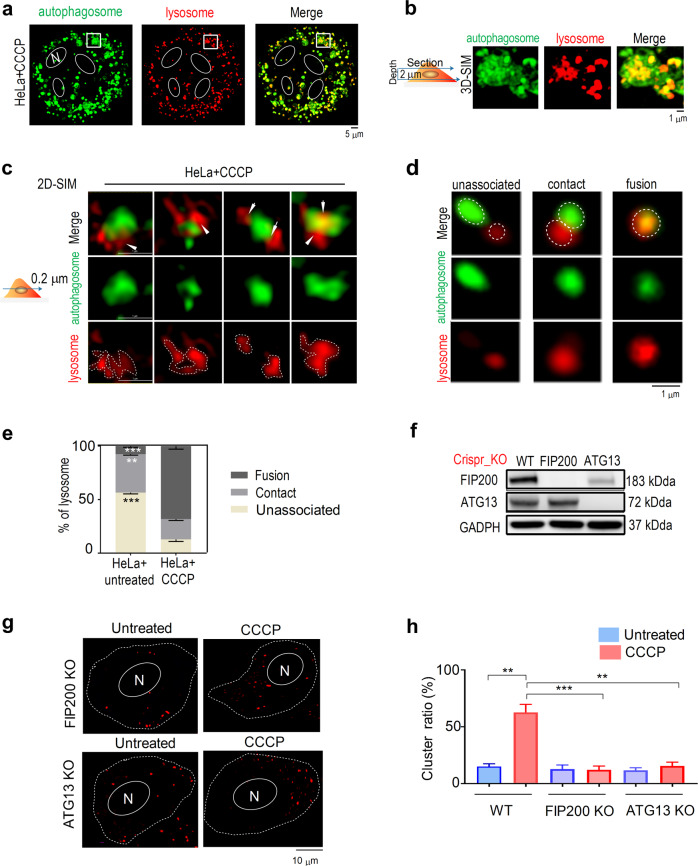
Lysosome clusters associate with autophagosomes. **a** Co-localization of autophagosomes and lysosomes in CCCP-treated HeLa cells was observed by SIM. Autophagosomes and lysosomes were stained with 100 nM DAPGreen and 200 nM LTR, respectively, for 30 min to allow the investigation of lysosome–autophagosome association. **b** 3D SIM image of the enlarged region indicated by white rectangles in **a**. **c** Representative 2D SIM images of several lysosomes associated with one autophagosome in each CCCP-treated HeLa cell. **d** Representative events of lysosomes unassociated, in contact, and fused with the autophagosomes. **e** Percentage of lysosomes unassociated, in contact, and fused with autophagosomes using the definition described in the text. **f** Western blot for detecting FIP200 and ATG13 protein expression in CRISPR-Cas9 gene-edited cells. **g** Lysosome puncta distribution in ATG13KO and FIP200KO HeLa cells with or without CCCP treatment. **h** The lysosome cluster ratio in WT, ATG13KO, and FIP200KO HeLa cells. Data in **e** and **h** are presented as mean ± SEM, *p < 0.05, **p < 0.01, ***p < 0.001.

Lysosomes fuse with autophagosomes to form autolysosomes for cargo degradation during autophagy [30, 31]. To confirm the potential interaction of lysosome clusters and autophagosomes, we stained lysosomes and autophagosomes with LTR and DAPG dyes, respectively (Fig. 2a), and observed a substantial overlap of lysosome clusters with autophagosomes (Fig. 2a-merge). As revealed by 3D SIM (Fig. 2b) and 2D SIM images of 200 nm deep sections (Fig. 2c) of CCCP-treated HeLa cells, multiple lysosomes bound to one autophagosome to form a cluster, which indicates that the formation of lysosome clusters is associated with autophagosomes. By contrast, in untreated HeLa cells, the overlap between lysosomes and autophagosomes decreased (Fig. 2d). To further confirm the reliability of DAPG dyes as autophagosome markers, we examined the colocalization of the DAPRed dyes, which is similar to DAPG except emitting red fluorescence, with LC3B-GFP puncta. DAPRed stained puncta colocalized very well with LC3B-GFP puncta in LC3B-GFP-expressing HeLa cells treated with CCCP and RM respectively (Supplementary Fig. S3). Moveover, we also used negative stain electron microscopy (EM) to confirm lysosomes clustering on autophagosome and remaining on autolysosome after the maturation of autophagosome (Supplementary Fig. S4). To analyze the details of lysosome–autophagosome association, we developed an algorithm to process SIM images of both organelles. Using a SIM resolution of approximately 200 nm, we defined lysosomes and autophagosomes as being unassociated when the distance between them is more than 200 nm, as in contact when the distance was less than 200 nm, and as fused when a yellow area emerged due to lysosome–autophagosome overlap (Fig. 2d). Using this definition, we quantified lysosome–autophagosome interaction before and after CCCP treatment (Fig. 2e), the results of which were consistent with Pearson’s *r* values for fusion obtained with the CellProfiler software (Supplementary Fig. S5) used to quantify overlap [29]. The greatest number of events involving lysosome–autophagosome fusion and contact were observed after CCCP treatment (Fig. 2e), which had previously facilitated lysosome clustering (Fig. 1e). That correlation and the morphologic overlap of lysosome clusters and autophagosomes (Fig. 2b, c) imply the important role of autophagosomes in recruiting lysosomes for clustering upon stimulation.

To verify the association of lysosome clusters with the formation of autophagosomes, endogenous ATG13 and FIP200 were respectively knocked out (KO) by using the CRISPR-Cas9 gene-editing tool (Fig. 2f) to block autophagosome formation [29]. The formation of lysosome clusters did not occur in ATG13KO and FIP200KO cells (Fig. 2g, h, respectively), which confirmed that lysosome clustering depends upon autophagosomes.

### VAMP8 regulates lysosome–autophagosome interaction

**Fig. 3:**
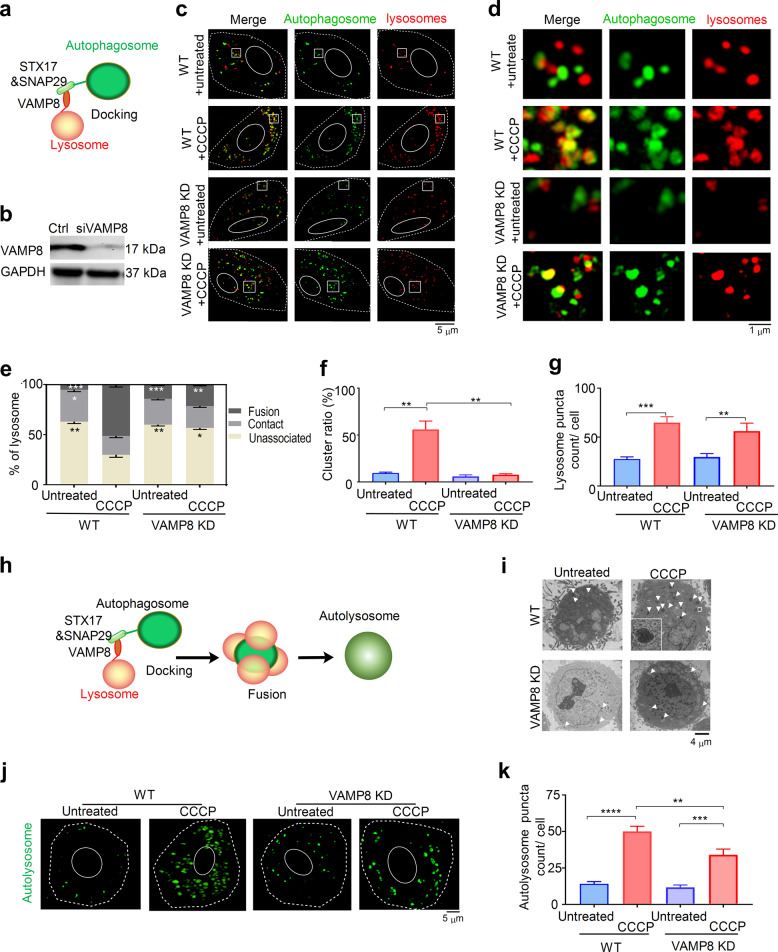
VAMP8 regulates lysosome–autophagosome interaction. **a** Schematic representation of VAMP8 in SNARE-mediated lysosome–autophagosome association. **b** Western blot for detecting VAMP8 protein expression in siRNA gene silencing cells. Cells were transfected with or without siVAMP8 for 48 h, and protein was collected for detecting VAMP8 protein expression. **c** Overlap of autophagosomes and lysosomes in VAMP8KD and WT HeLa cells with or without CCCP treatment. **d** Close-up views of lysosome–autophagosome associations for the enlarged region indicated by white rectangles in **c**. **e** Percentile distribution of lysosomes unassociated, in contact, and fused with autophagosomes. **f** Ratio of clustered lysosome clusters to the total number of lysosomes and (**g**) lysosome count in WT and VAMP8KD HeLa cells with or without CCCP treatment. **h** Schematic representation of VAMP-induced lysosome clustering on an autophagosome. **i** Autolysosome formation observed by negative stain EM. **j** SIM image and **k** count of autolysosome formations in WT and VAMP8KD HeLa cells. Autolysosomes were stained with 100 nM of DALGreen at 37 °C for 30 min, followed by CCCP for 12 h to induce autophagy and then observed under SIM. Data in **e**, **f**, **g**, and **k** are presented as mean ± SEM, *p < 0.05, **p < 0.01, ***p < 0.001, ****p < 0.0001.

SNARE proteins are involved in the fusion of lysosomes with autophagosomes [32]. Among the relevant SNAREs, VAMP8 is located on the lysosomal membrane [33] (Fig. 3a). Thus, we hypothesized that it plays an important role in directing lysosomes to autophagosomes. To test this possibility, knockdown (KD) of endogenous VAMP8 was performed by siRNA to downregulate the expression of VAMP8 (Fig. 3b). Compared to wild-type (WT) cells, upon CCCP stimulation, the percentage of lysosome fusion and association with autophagosomes decreased in VAMP8KD cells (Fig. 3c–e). At the same time, the formation of lysosome clusters decreased in VAMP8KD cells (Fig. 3f) without altering the number of lysosomes (Fig. 3g). The importance of VAMP8 was further confirmed with another autophagy inducer, RM [26, 27]. As shown in Supplementary Fig. S6, knockdown of VAMP8, no significant difference was found in the percentage of lysosome fusion and association with autophagosomes with and without RM treatment. Our results suggest that VAMP8 plays a key role in lysosome–autophagosome association and lysosome clustering (Fig. 3h).

To verify the effect of reduced lysosome clustering caused by VAMP8KD on autophagosome-lysosome fusion, we used negative stain EM (Fig. 3i) [34] and an autolysosome dye to stain cells for SIM experiments (Fig. 3j) in order to investigate the formation of autolysosomes. The results indicated that VAMP8 silencing also decreased the formation of autolysosomes (Fig. 3k). By contrast, clustered lysosomes showed a higher probability of overlap with damaged mitochondria (Supplementary Fig. S7). Taken together, the formation of lysosome clusters may be important for the prefusion state between lysosomal and autophagosomal membranes.

### Phosphorylation of VAMP8 hinders lysosome–autophagosome fusion

Next, we investigated the mechanisms that control the fusion of lysosome clusters with autophagosomes. Previously, we studied the SNARE proteins VAMP8–Syntaxin17–SNAP29 and their roles in lysosome–autophagosome fusion [33], and we also investigated the effect of phosphorylation of VAMP8 by protein kinase C (PKC) in secretory vesicle fusion [35]. We thus hypothesized that phosphorylation of VAMP8 could reduce lysosome–autophagosome fusion, whereas its dephosphorylation would resume normal autophagic flux.

**Fig. 4:**
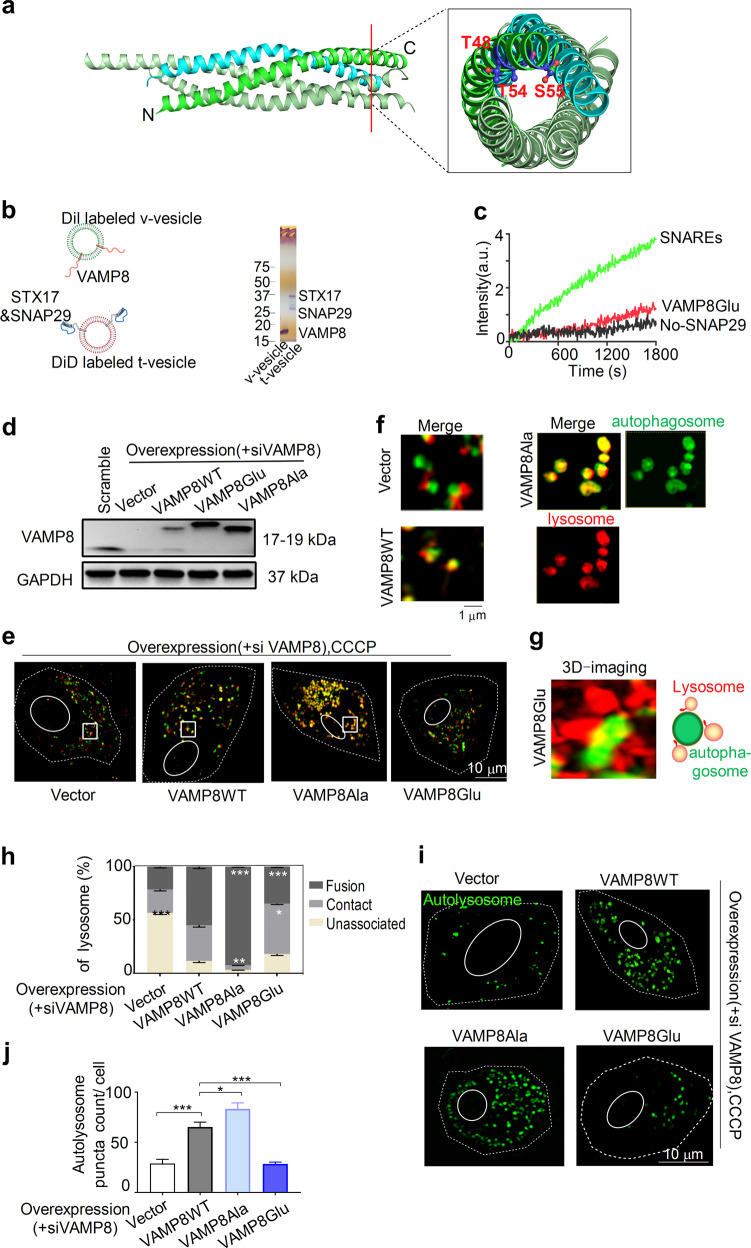
Phosphorylation of VAMP8 hinders lysosome–autophagosome fusion. **a** Crystal structure of VAMP8 in a SNARE complex (PDB: 4WY4) involved in autophagy. T48, T54, and S55 are the three phosphorylation sites of VAMP8 in the SNARE domain. **b** Schematic representation of vesicle reconstitution for mimicking lysosome–autophagosome fusion. SDS-PAGE gel image of proteoliposomes indicating the reconstitution of SNARE proteins. **c** Reduced lipid mixing was observed for VAMP8Glu mutants (i.e., T48E, T54E, and S55E). **d** Western-blot assay for the detection of endogenous and induced VAMP8 and its mutants. The gel band shift between endogenous and induced VAMP8 was caused by the FLAG tag. **e** Overlap of autophagosomes and lysosomes in CCCP-treated VAMP8KD, WT rescue, VAMP8Ala mutants (i.e., T48A, T54A, S55A), and VAMP8Glu mutants cells. Cells were transfected with or without siVAMP8 and mutant vector for 48 h. Autophagosomes and lysosomes were stained with 100 nM DAPGreen and 200 nM LTR, respectively, for 30 min to allow the investigation of lysosome–autophagosome association using SIM. **f** Close-up views of SIM images of fusion in vector, VAMP8WT, VAMP8Ala mutants cells for the enlarged region indicated by white rectangles in **e**. **g** Representative 3D SIM images of lysosomes in association with autophagosomesin VAMP8Glu mutants cells. **h** Percentile distribution of lysosomes unassociated, in contact, and fused with autophagosomes in CCCP-treated VAMP8KD, WT rescue, VAMP8Ala mutants, and VAMP8Glu mutants HeLa cells. **i** SIM images and **j** count of autolysosome formations in VAMP8KD, WT rescue, VAMP8Ala mutants, and VAMP8Glu mutants HeLa cells with CCCP treatment. Data in **h** and **j** are presented as mean ± SEM, *p < 0.05, **p < 0.01, ***p < 0.001, ****p < 0.0001.

To test this hypothesis, we mutated the three phosphorylation sites of VAMP8 to glutamic acid (i.e., VAMP8Glu, T48E, T54E, and S55E) in order to mimic VAMP8 phosphorylation (Fig. 4a) and studied SNARE-mediated fusion of vesicle membranes using an ensemble lipid mixing assay in vitro [36, 37] (Fig. 4b, c). We found that VAMP8Glu reduced the fusion of proteoliposomes reconstituted with VAMP8 and Syntaxin17–SNAP29. To confirm our hypothesis at the cellular level, we mutated three phosphorylation sites of VAMP8 to alanine (i.e., VAMP8Ala, T48A, T54A, and S55A) in order to prevent all three sites from being phosphorylated in vivo (Fig. 4d), which was confirmed by a gel band shift between the induced VAMP8WT and VAMP8Ala. Fusion events with VAMP8Ala increased (Fig. 4e, f, h). Moreover, VAMP8Glu also induced a considerable amount of lysosome–autophagosome contacts (Fig. 4g), whereas their fusion decreased (Fig. 4e, h). We monitored the formation of autolysosomes using SIM of DAPG-stained samples (Fig. 4i, j) and found that their formation also increased inVAMP8Ala cells but decreased in VAMP8Glu cells. We also confirmed this observation with another autophagy indicator, LC3B with CCCP-treatment (Supplementary Fig. S8) or in RM-treated HeLa cells stably expressing GFP-LC3B (Supplementary Fig. S9). Our data suggest that VAMP8 phosphorylation plays an important role in regulating lysosome–autophagosome fusion. Furthermore, we observed that both autophagosome-lysosome fusion and the formation of lysosome clusters that are associated with autolysosomes increased in PKC inhibitor-treated HeLa cells (Supplementary Fig. S10).

### Phosphorylation of VAMP8 regulates temozolomide (TMZ) resistance of HeLa cells

**Fig. 5:**
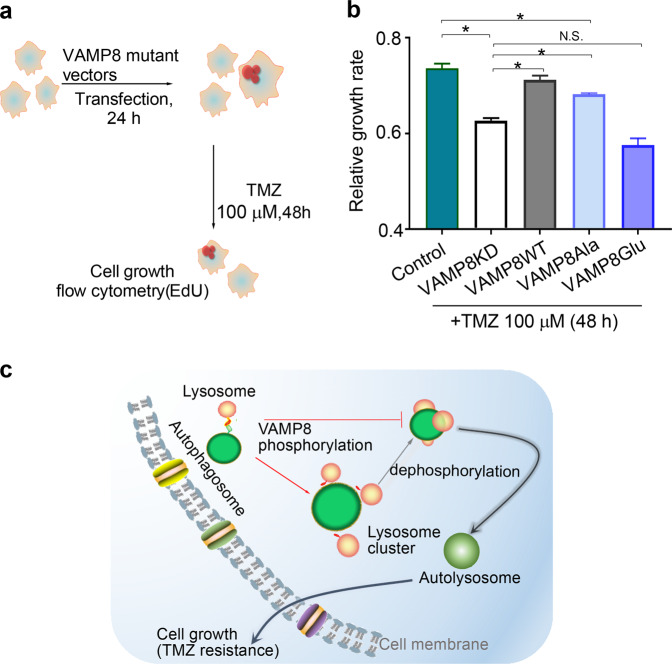
Phosphorylation of VAMP8 regulates temozolomide (TMZ) resistance of HeLa cells. **a** Schema of TMZ resistance measurement and **b** Quantitative data in HeLa and VAMP8KD HeLa cells re-expressed by empty-vector, WT, VAMP8Ala, and VAMP8Glu. **c** Schematic representation of a VAMP8-regulated lysosome cluster on autophagosomes. VAMP8 controls the formation of lysosome clusters associated with autophagosomes, whereas its phosphorylation inhibits fusion for autophagosome maturation. Data in (**b**) are presented as mean ± SEM, *p < 0.05.

Because autophagy is known to determine cell fate [38], we studied the physiological and pathological role of VAMP8 phosphorylation. We found that knockdown of VAMP8 decreased TMZ resistance (Fig. 5a, b), similar to a previous study that showed that VAMP8 silencing could reduce TMZ resistance in glioma cells [39]. Compared to the phosphorylation mimicking mutant of VAMP8 (VAMP8Glu), the phosphorylation defective mutant of VAMP8 (VAMP8Ala) showed an enhancement in TMZ resistance. Together, these results suggest that VAMP8 phosphorylation plays an important role in TMZ resistance by influencing autophagic flux.

## Conclusion

To achieve efficient fusion upon stimulation, a possible mechanism is the formation of a prefusion state that undergoes fusion upon stimulation [40, 41]. The precise mechanism of membrane fusion involved in autophagosome maturation remains unknown. Here, we report that lysosomes are clustered on autophagosomes. This prefusion “architecture” could possibly increase the efficiency of fusion by providing multiple fusion sites. Moreover, this prefusion architecture implies that multiple lysosomes could fuse with one autophagosome for efficient cargo degradation.

Because SNARE proteins are known to induce spontaneous membrane fusion, other factors likely play inhibitory roles in the regulation of fusion [20]. At the same time, because autophagy is minimized under normal cellular conditions, a “brake” for spontaneous SNARE-mediated membrane fusion is essential for suppressing autophagic flux under normal conditions. Here, we observed that VAMP8 plays an important role in the formation of lysosome clusters, and its phosphorylation at the C-terminal end reduces fusion activity. Therefore, VAMP8 phosphorylation contributes to the formation of unfused lysosome clusters on autophagosomes and works as a switch between membrane contact and fusion to control the autophagic flux (Fig. 5c). Conversely, upon stimulation, dephosphorylation for the formation of new fully zippered SNAREs may resume lysosome–autophagosome fusion to promote autophagy. In a stress condition, the preassembly of multiple lysosomes around one autophagosome could increase the fusion probability upon dephosphorylation of VAMP8. Thus, VAMP8 phosphorylation works as a dual-regulator, suggesting a regulatory mechanism of lysosomal fusion with the autophagosome through post-translational modification.

VAMP8 was found to be an oncoprotein by facilitating glioma cell growth and chemotherapy resistance to TMZ [39]. Our results suggest a molecular mechanism that may explain the pathological role of VAMP8. Moreover, dephosphorylation of VAMP8 could be responsible for tumor malignancy, and VAMP8 phosphorylation could play an important role on regulating cancer cell survival. Furthermore, because autophagy has been proposed as a prosurvival mechanism essential for drug resistance [42], enhancing VAMP8 phosphorylation to block autophagosome maturation could be a strategy for reducing drug resistance.

## Materials and methods

### Materials

LysoTracker Red (LTR, L12492) and MitoTracker Green (MTG, M7514) were obtained from Invitrogen (Carlsbad, CA), whereas autophagosome dye (DAPGreen, NY561) and autolysosome dye (DALGreen, PF727) were obtained from Dojindo Laboratories (Kumamoto, Japan). Carbonyl cyanide *m*-chlorophenylhydrazone (045200), rapamycin (12921), and oligomycin A (41105) were obtained from Thermo Fisher Scientific (Grand Island, NY). Penicillin–streptomycin (15140163, 10,000 units/mL), fetal bovine serum (FBS; 26140079), horse serum (26050-088), Dulbecco’s modified Eagle’s medium (DMEM; 11965118), F12K cell media (21127-022), phenol-free medium (1894117), and other cell culture reagents were obtained from Gibco BRL (Grand Island, NY). Primary and secondary antibodies used were GAPDH (5174, Cell Signaling Technology, Danvers, MA), FIP200 (12436, Cell Signaling Technology), ATG13 (13273, Cell Signaling Technology), VAMP8 (ab76021, Abcam, Cambridge, MA), HRP-linked anti-rabbit IgG (7074, Cell Signaling Technology), siRNAVAMP8 (VAMP8-HSS112731, 1299001, Invitrogen, Carlsbad, CA), and silencer negative control siRNA (AS020K25, Ambion, Austin, TX), Coupa-lyso probe was grifted from Zijian Guo lab (Nanjing University, Nanjing, China).

### Mammalian cell culture

HeLa cells were donated by: Dr. Carolyn M. Price (University of Cincinnati, OH), A549 cells by Dr. Jagjit S. Yadav (University of Cincinnati), and SLC-80 cells isolated from healthy human fibroblasts by Dr. Taosheng Huang (Cincinnati Children’s Hospital, Cincinnati, OH). HeLa cells, A549 cells, and SLC-80 cells were cultured in DMEM supplemented with 10% FBS and penicillin–streptomycin (100 units/mL) in a 5% CO_2_ humidified incubator at 37 °C. PC12 cells donated by Dr. Kai Zhang (University of Illinois at Urbana–Champaign, IL) were cultured in F12K medium supplemented with 15% horse serum, 2.5% FBS, and penicillin-streptomycin (100 units/mL) in a 5% CO_2_-humidified incubator at 37 °C.

### Live-cell labeling and sample preparation for imaging

Cells were split and cultured in a glass-bottom microwell dish and incubated with 2 mL of DMEM supplemented with 10% FBS for 24 h, followed by 10.0 μM CCCP, 0.5 μM RM, 1 μM O/A or EBSS medium for 12 h. After treatment, the cells were washed 3 times with prewarmed free DMEM and incubated with 200 nM of LTR for lysosome staining at 37 °C for 30 min. Afterward, the cells were again washed with free DMEM 3 times and incubated in a phenol-free medium and observed under confocal laser scanning microscopy or SIM (Nikon, Tokyo, Japan). For the autolysosome or autophagosome-lysosome co-staining assays, cells were exposed to 1 mL of free medium containing 100 nM of DALGreen or DAPGreen at 37 °C for 30 min, after which the supernatant was discarded and washed 3 times with prewarmed free DMEM, followed by CCCP or vehicle treatment for 12 h to induce autophagy. After treatment, the cells were incubated with 200 nM of LTR for lysosome staining to allow the investigation of lysosome–autophagosome interaction.

### VAMP8 silencing by siRNA and mutant vector

Scrambled siRNA and siVAMP8 were transiently delivered by Lipofectamine 3000 (Invitrogen, Carlsbad, CA) into HeLa cells according to the manufacturer’s protocol. FLAG-VAMP8 vector was purchased from Addgene (45912) (Watertown, MA). The triple mutant construct was created by using the Q5 Site-Directed Mutagenesis Kit (E0552S, New England Biolabs, Ipswich, MA) following the manufacturer’s protocol. The primers for amplifying VAMP8Glu were VAMP8 (Glu)-F: gaagaagagcacttcaagacgacatcg and VAMP8 (Glu)-R: ggcttccagatcctcttccttgttgcggagatgttcca, whereas those for amplifying and VAMP8Ala were VAMP8 (ALa)-F: gcagctgagcacttcaagacgacatcg and VAMP8 (ALa)-R: ggcttccagatcctctgccttgttgcggagatgttcca.

### Confocal imaging

The images were obtained using an LSM-710 confocal laser scanning microscope (Carl Zeiss, Inc., Germany) equipped with a 63×/1.49 numerical aperture oil immersion objective lens and analyzed with ZEN 2012 (Carl Zeiss, Inc., Germany) and ImageJ software (National Institutes of Health).

### SIM super-resolution microscopy imaging

Super-resolution images were acquired on a commercial SIM microscope (Nikon). Images were obtained at 512 × 512 using Z-stacks with a step size of 0.2 μm. For 3D SIM, more than 10 stacks with a step size of 0.2 μm were obtained with a total depth of 2 μm. All fluorescence images were analyzed, and their backgrounds were subtracted with ImageJ software (National Institutes of Health).

### CRISPR–Cas9-mediated KO of FIP200 and ATG13 in HeLa cells

The pX458 plasmid pSpCas9(BB)-2A-GFP (Addgene, Watertown, MA) was used as the cloning backbone for expressing sgFIP200 and sgATG13. Two complementary oligomers for each sgRNA were denatured, annealed, and ligated into linearized pX458 vector digested by BbsI (New England Biolabs, Ipswich, MA). Empty constructs and pooled pX458-sgRNA were transiently transfected into HeLa cells using Lipofectamine 3000 (Invitrogen, Carlsbad, CA). After 48 h, the transfected cells were sorted according to the fluorescence of GFP as a reporter using a BD FACSAria III cytometer (BD Biosciences, Franklin Lakes, NJ). Sorted individual cells were cultured in a 96-well plate and subjected to western blot analyses. At least three different clones were pooled for functional experiments. The sgRNA sequences targeting FIP200 and ATG13 were taken from past studies that involved using the CRISPR-Cas9 library (Sanjana et al., 2014; Wang et al., 2014). sgRNA sequences of FIP200 and ATG13 were:

sgFIP200: CAGGTGCTGGTGGTCAATGG;

sgATG13-1: TCACCCTAGTTATAGCAAGA;

sgATG13-2: CAGTCTGTTGTACACCGTGT;

sgATG13-3: GACTGTCCAAGTGATTGTCC.

### Generation of HeLa^LC3B-GFP^ cells

Stable overexpression of LC3B-GFP was achieved by lentivirus transfection into WT HeLa cells followed by sorting of GFP under flow cytometry. Lentivirus was generated by the transfection of psPAX2, pMD2.G and lentiviral plasmids into HEK293T cells.

### Immunofluorescence (IF) staining

For immuno-staining of LAMP2, cells cultured on glass coverslips were fixed with methanol at 4 °C for 10 min and blocked with PBS containing 5% goat serum for 1 h. Coverslips were incubated overnight at 4 °C with primary antibody prepared in PBS containing 5% goat serum. After 3 washes with PBS, coverslips were incubated with Rhodamine-conjugated anti-rat for 2 h at room temperature. Cells were washed 3 times with PBS under low light conditions and stained with 1 μg/ml DAPI. All images were generated on Zeiss LSM 710 confocal laser scanning microscope.

### Western blotting

Cells were cultured in plates 3.5 cm in diameter (80–90% confluence), washed by PBS buffer, and lysed for 15 min on ice using radioimmunoprecipitation assay buffer (C2978, Sigma–Aldrich, St. Louis, MO) containing an antiprotease mix (PI78415, Thermo Scientific, Waltham, MA). Protein concentration was measured by bicinchoninic acid (23225, Thermo Scientific). To alleviate the effect of membrane overexposure, serial dilution of cell lysates starting from a 50 μg sample over at least 10 protein dilutions was applied to generate a standard curve of band density for each protein target. Quantification of band density of each target was achieved by ImageJ software. To assure that the densitometric data for all of the target proteins will be within the linear quantitative range, 15 μg of proteins were subjected to SDS-PAGE and immunoblotting.

### Electron microscopy

The cells were removed from a Petri dish with a flat scraper and collected by centrifugation at 1000 g. The sample was fixed using the Karnovsky method, then fixed in 2% osmium tetroxide and dehydrated in a series of graded ethanol and embedded in Araldite. Ultra-thin sections were cut using an LKB glass knife (Leica EM UC7, Buffalo Grove, IL) and collected on a Formvar coated grid. The sections of 90 nm thick were stained with 2% uranyl acetate & lead citrate, and evaluated in a transmission electron microscope (Hitachi, H-7650, V01.07, Tokyo, Japan) with an acceleration voltage of 80 kV.

### Temozolomide (TMZ) resistance

TMZ resistance was evaluated by monitoring cell proliferation. Cells were cultured with 5-ethynyl-2′-deoxyuridine (EdU) treatment (100 nM) for 4 h. The formalin-fixed cells were stained with Tris (100 mM), CuSO4 (1 mM), fluorescent-488 azide (100 μM), ascorbic acid (50 mM), and DAPI (1 μg/ml) for 30 min. Three independent replicates for stained cells were analyzed by FACScan (BD Biosciences) and counted for statistical analysis.

### Ensemble lipid mixing assays

Protein-reconstituted *t*- and *v*-SNARE proteoliposomes were mixed at a molar ratio of 1:1. The ensemble lipid mixing experiments were performed with DiI donor-dye and DiD acceptor-dye labeled *t*- and *v*-SNARE proteoliposomes, respectively, similar to previously published work [35]. Briefly, donor dyes were excited with a 530 nm laser light, and emission fluorescence intensity was monitored at 570 and 670 nm. Lipid mixing was measured as the fluorescence emission at 670 nm of DiD acceptor dyes arising from FRET upon the excitation of DiI dyes with the 530 nm light. Fluorescence emission was recorded with a Varian Cary Eclipse model fluorescence spectrophotometer using a quartz cell of 100 μL with a 5 mm path length. All lipid mixing measurements were performed at 35 ± 2 °C.

### Data analysis

Statistical analysis was performed with MATLAB 2017a (MathWorks) or Prism 7 (GraphPad). The statistical comparison of results was performed with a Student’s *t* test (Mann–Whitney) or ANOVAs with levels of significance set at *p < 0.05, **p < 0.01, ***p < 0.001, and ****p < 0.0001. Data are presented as mean ± SEM.

### Statistics and reproducibility

Each experiment was repeated three times independently with similar results. All images shown are representative results from biological replicates

## Supplementary information

Supprting Figures

## Data Availability

The datasets generated and/or analyzed during the current study are available from the corresponding authors on reasonable request.

## References

[1] Lawrence RE, Zoncu R (2019). The lysosome as a cellular centre for signalling, metabolism and quality control. Nat Cell Biol.

[2] Saftig P, Klumperman J (2009). Lysosome biogenesis and lysosomal membrane proteins: trafficking meets function. Nat Rev Mol Cell Biology.

[3] Li P, Gu M, Xu H (2018). Lysosomal ion channels as decoders of cellular signals. Trends Biochemical Sci.

[4] Platt FM, d’Azzo A, Davidson BL, Neufeld EF, Tifft CJ (2018). Lysosomal storage diseases. Nat Rev Dis Prim.

[5] Yim WW-Y, Mizushima N (2020). Lysosome biology in autophagy. Cell Discov.

[6] Kawabata T, Yoshimori T (2020). Autophagosome biogenesis and human health. Cell Discov.

[7] Ba Q, Raghavan G, Kiselyov K, Yang G (2018). Whole-cell scale dynamic organization of lysosomes revealed by spatial statistical analysis. Cell Rep..

[8] Sugiura A, Mattie S, Prudent J, McBride HM (2017). Newly born peroxisomes are a hybrid of mitochondrial and ER-derived pre-peroxisomes. Nature.

[9] Wong YC, Ysselstein D, Krainc D (2018). Mitochondria–lysosome contacts regulate mitochondrial fission via RAB7 GTP hydrolysis. Nature.

[10] Hsieh C-W, Yang WY (2019). Omegasome-proximal PtdIns (4, 5) P 2 couples F-actin mediated mitoaggregate disassembly with autophagosome formation during mitophagy. Nat Commun.

[11] Delarue M, Brittingham GP, Pfeffer S, Surovtsev IV, Pinglay S, Kennedy KJ (2018). mTORC1 controls phase separation and the biophysical properties of the cytoplasm by tuning crowding. Cell.

[12] Sigal YM, Zhou R, Zhuang X (2018). Visualizing and discovering cellular structures with super-resolution microscopy. Science.

[13] Hell SW, Wichmann J (1994). Breaking the diffraction resolution limit by stimulated emission: stimulated-emission-depletion fluorescence microscopy. Opt Lett.

[14] Gustafsson MGL (2000). Surpassing the lateral resolution limit by a factor of two using structured illumination microscopy. J Microsc.

[15] Rust MJ, Bates M, Zhuang X (2006). Sub-diffraction-limit imaging by stochastic optical reconstruction microscopy (STORM). Nat Methods.

[16] Betzig E, Patterson GH, Sougrat R, Lindwasser OW, Olenych S, Bonifacino JS (2006). Imaging intracellular fluorescent proteins at nanometer resolution. Science.

[17] Diao J, Grob P, Cipriano DJ, Kyoung M, Zhang Y, Shah S (2012). Synaptic proteins promote calcium-triggered fast transition from point contact to full fusion. eLife.

[18] Hui E, Johnson CP, Yao J, Dunning FM, Chapman ER (2009). Synaptotagmin-mediated bending of the target membrane is a critical step in Ca2+-regulated fusion. Cell.

[19] Martens S, Kozlov MM, McMahon HT (2007). How synaptotagmin promotes membrane fusion. Science.

[20] Brunger AT, Choi UB, Lai Y, Leitz J, Zhou Q (2018). Molecular mechanisms of fast neurotransmitter release. Annu Rev Biophysics.

[21] Chen Q, Shao X, Hao M, Guan R, Tian Z, Li M (2020). Quantitative analysis of interactive behavior of mitochondria and lysosomes using structured illumination microscopy. Biomaterials.

[22] Qiu K, Du Y, Liu J, Guan J-L, Chao H, Diao J (2020). Super-resolution observation of lysosomal dynamics with fluorescent gold nanoparticles. Theranostics.

[23] Chen Q, Jin C, Shao X, Guan R, Tian Z, Wang C (2018). Super‐resolution tracking of mitochondrial dynamics with an Iridium (III) Luminophore. Small.

[24] Fang H, Geng S, Hao M, Chen Q, Liu M, Liu C (2021). Simultaneous Zn2+ tracking in multiple organelles using super-resolution morphology-correlated organelle identification in living cells. Nat Commun.

[25] Chen Q, Fang H, Shao X, Tian Z, Geng S, Zhang Y (2020). A dual-labeling probe to track functional mitochondria–lysosome interactions in live cells. Nat Commun.

[26] Zhang X, Chen W, Gao Q, Yang J, Yan X, Zhao H (2019). Rapamycin directly activates lysosomal mucolipin TRP channels independent of mTOR. PLoS Biol.

[27] Shao X, Chen Q, Hu L, Tian Z, Liu L, Liu F (2020). Super-resolution quantification of nanoscale damage to mitochondria in live cells. Nano Res.

[28] Abada A, Levinzaidman S, Porat Z, Dadosh T, Elazar Z (2017). SNARE priming is essential for maturation of autophagosomes but not for their formation. Proc Natl Acad Sci USA.

[29] Chen Q, Shao X, Tian Z, Chen Y, Mondal P, Liu F (2019). Nanoscale monitoring of mitochondria and lysosome interactions for drug screening and discovery. Nano Res.

[30] Wang Y, Li L, Hou C, Lai Y, Long J, Liu J (2016). SNARE-mediated membrane fusion in autophagy. Semin Cell Developmental Biol.

[31] Youle RJ, Narendra DP (2011). Mechanisms of mitophagy. Nat Rev Mol Cell Biol.

[32] Nair U, Jotwani A, Geng J, Gammoh N, Richerson D, Yen W-L (2011). SNARE proteins are required for macroautophagy. Cell.

[33] Diao J, Liu R, Rong Y, Zhao M, Zhang J, Lai Y (2015). ATG14 promotes membrane tethering and fusion of autophagosomes to endolysosomes. Nature.

[34] Lin J, Huang Z, Wu H, Zhou W, Jin P, Wei P (2014). Inhibition of autophagy enhances the anticancer activity of silver nanoparticles. Autophagy.

[35] Malmersjö S, Di Palma S, Diao J, Lai Y, Pfuetzner RA, Wang AL (2016). Phosphorylation of residues inside the SNARE complex suppresses secretory vesicle fusion. EMBO J.

[36] Diao J, Li L, Lai Y, Zhong Q (2017). In vitro reconstitution of autophagosome–lysosome fusion. Methods Enzymol.

[37] Tian Z, Gong J, Crowe M, Lei M, Li D, Ji B (2019). Biochemical studies of membrane fusion at the single-particle level. Prog Lipid Res.

[38] Green DR, Levine B (2014). To be or not to be? How selective autophagy and cell death govern cell fate. Cell.

[39] Chen Y, Meng D, Wang H, Sun R, Wang D, Wang S (2015). VAMP8 facilitates cellular proliferation and temozolomide resistance in human glioma cells. Neuro Oncol.

[40] Zhou Q, Zhou P, Wang AL, Wu D, Zhao M, Sudhof TC (2017). The primed SNARE–complexin–synaptotagmin complex for neuronal exocytosis. Nature.

[41] Zhou Q, Lai Y, Bacaj T, Zhao M, Lyubimov AY, Uervirojnangkoorn M (2015). Architecture of the synaptotagmin–SNARE machinery for neuronal exocytosis. Nature.

[42] Desantis V, Saltarella I, Lamanuzzi A, Mariggio MA, Racanelli V, Vacca A (2018). Autophagy: a new mechanism of prosurvival and drug resistance in multiple myeloma. Transl Oncol.

