# Supplementary material for: Prefused lysosomes cluster on autophagosomes regulated by VAMP8
Source: Cell Death Dis. 2021 Oct 13;12(10):939. doi: 10.1038/s41419-021-04243-0 (PMC8514493; doi:10.1038/s41419-021-04243-0)
Supplement: Supplementary file 1 — Supprting Figures [file 41419_2021_4243_MOESM1_ESM.docx]

Supporting Information

**Prefused lysosomes cluster on autophagosomes regulated by VAMP8**

Qixin Chen, Mingang Hao, Lei Wang, Linsen Li, Yang Chen, Xintian Shao, Zhiqi Tian, R.A. Pfuetzner, Qing Zhong, Axel T. Brunger, Jun-Lin Guan, Jiajie Diao


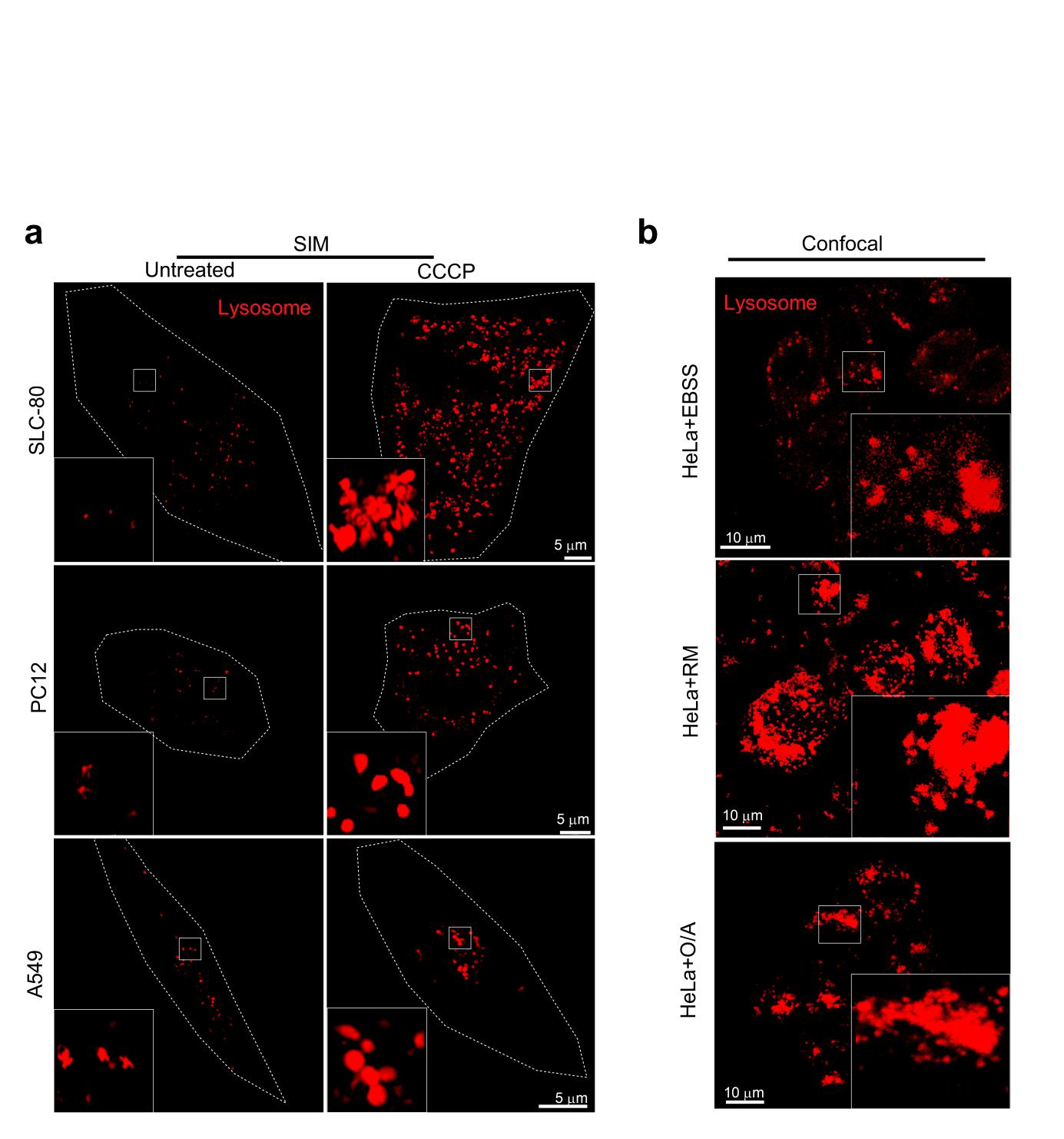


**Supplementary Figure S1** Imaging of lysosome clusters in other cell lines using structured illumination microscopy (SIM) and confocal microscopy. **a** The SLC-80, PC12, and A549 cell lines were treated with or without CCCP for 12 h and stained with LTR for 30 min before imaging using SIM. **b** EBSS-, RM-, and O/A-treated HeLa cells were stained with LTR for 30 min and imaged using a confocal microscope for the formation of lysosome clusters (insets).


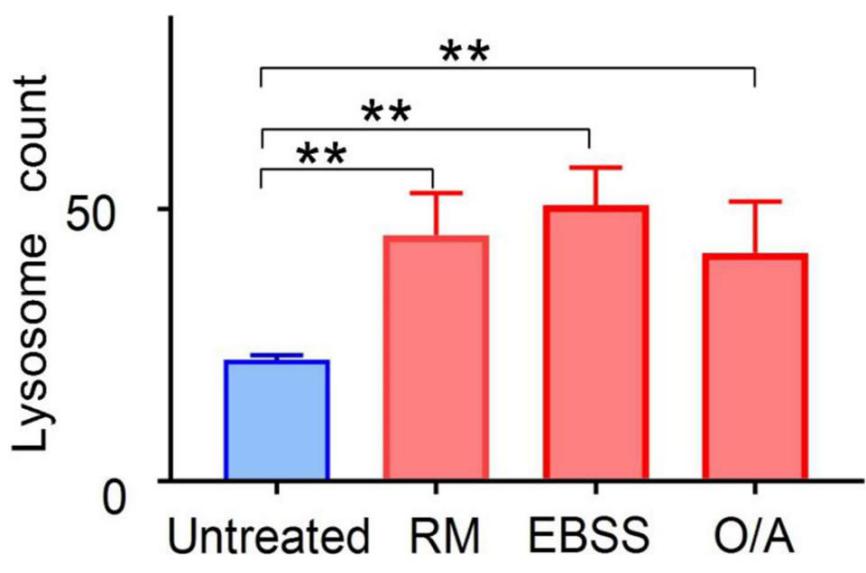


**Supplementary Figure S2** Lysosome count in HeLa cells treated with RM, EBSS, and O/A treatment. Data are presented as mean ± SEM, ***p* < 0.001.


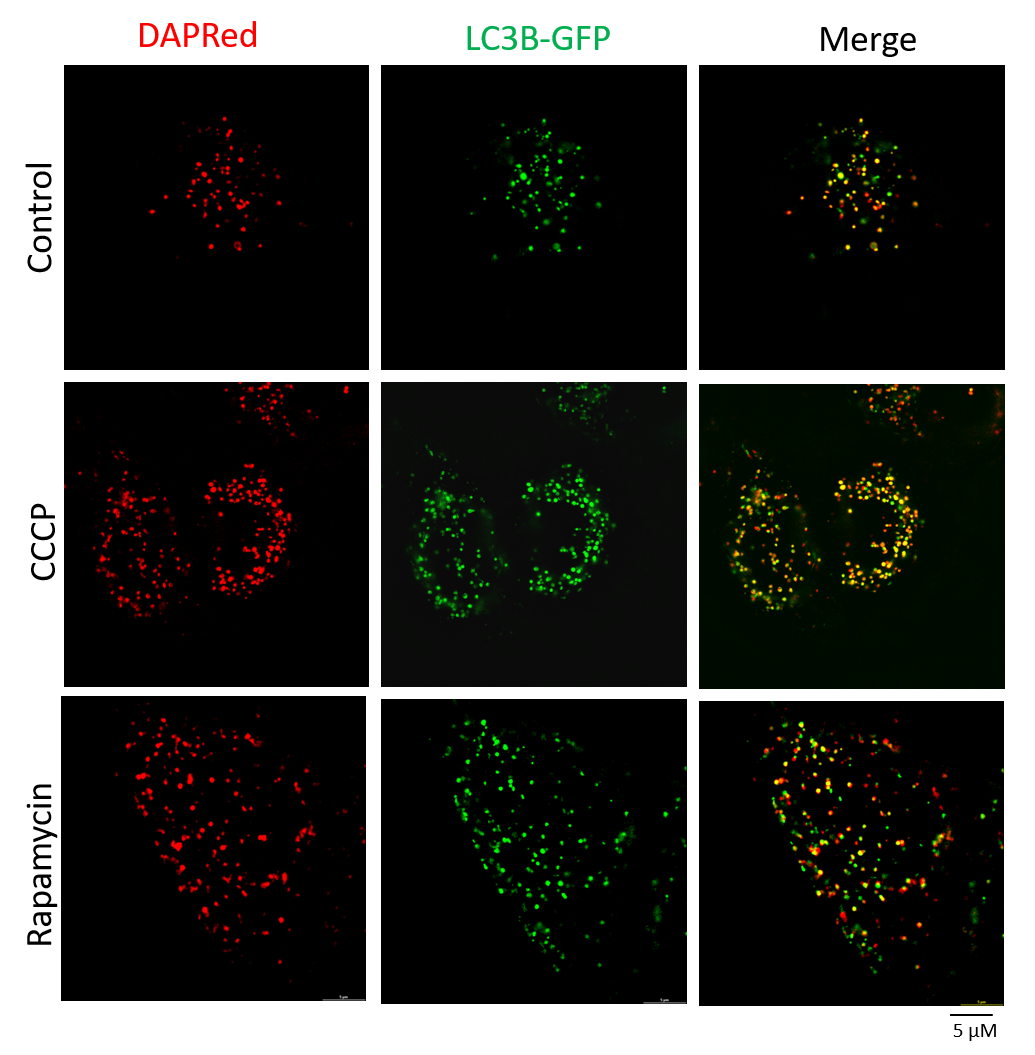


**Supplementary Figure S3** DAPRed is a competent autophagosome marker. LC3B-GFP-expressing HeLa cells treated without or with 10 µM CCCP or 500 nM RM for 12 h prior to be stained with 100 nM DAPRed for 30 min, and then observed by SIM.

###
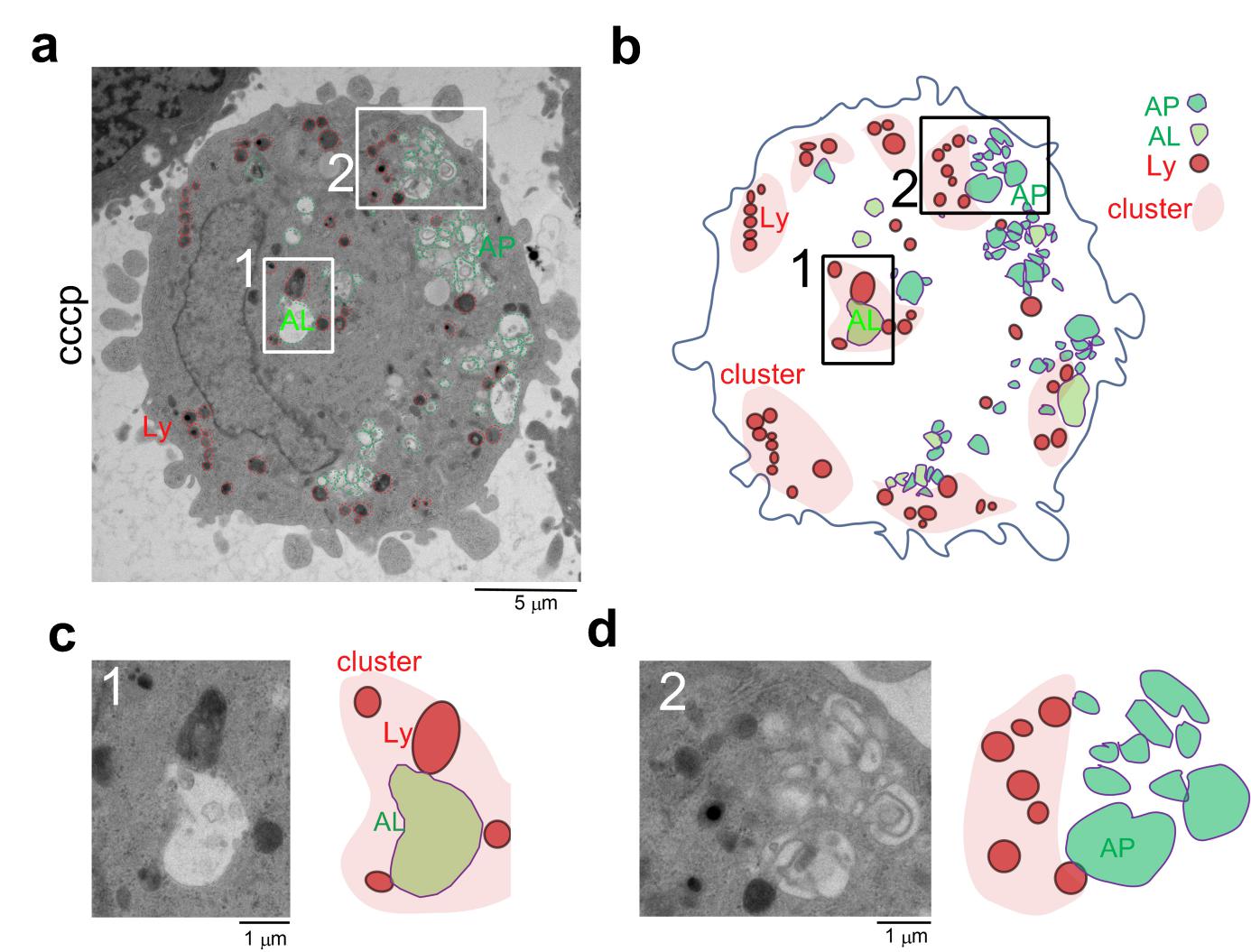


**Supplementary Figure S4** Transmission electron microscopy (TEM) reveals lysosomes interact with autophagosome/autolysosome in mitophagy. **a** TEM image of CCCP-treated HeLa cell and **b** the corresponding morphological recognition. **c** Enlarged region 1 indicates lysosome clustering on an autolysosome after fusion and **d** enlarged region 2 shows the lysosome clustering on an autophagosome before fusion.


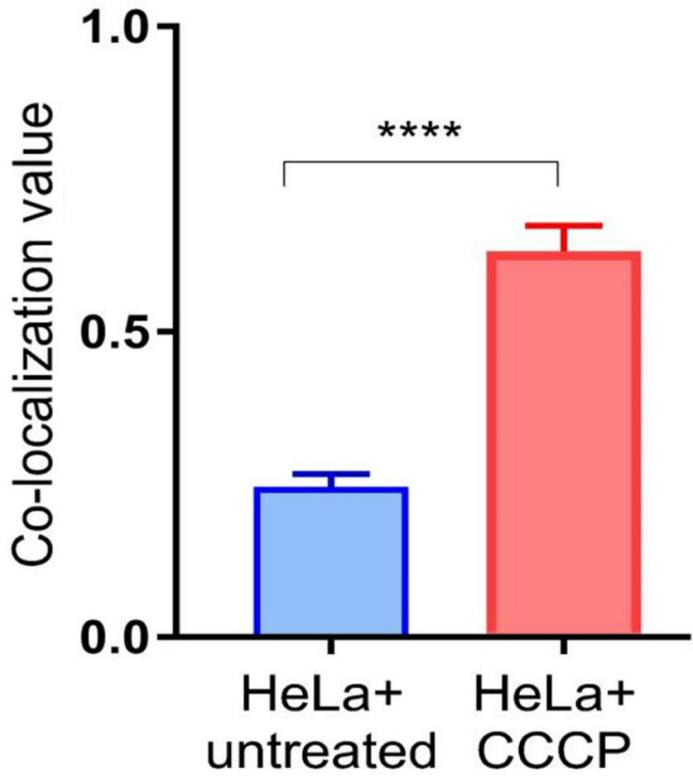


**Supplementary Figure S5** Joint localization of autophagosomes and lysosomes in HeLa cells with or without CCCP treatment. The overlap of autophagosomes and lysosomes was analyzed using the CellProfiler software (Carpenter Lab, Broad Institute of Harvard and MIT). Data are presented as mean ± SEM, *****p* < 0.0001.


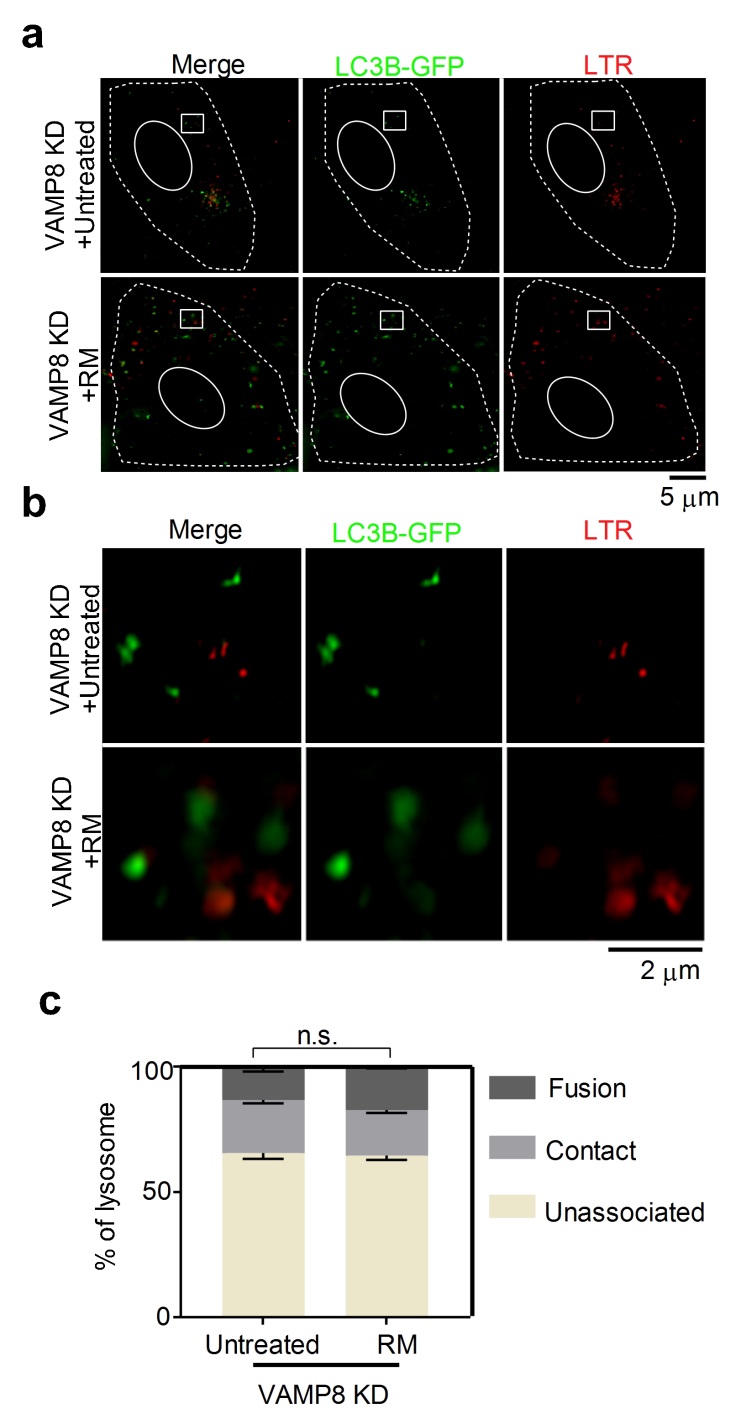


**Supplementary Figure S6** The interaction between autophagosomes and lysosomes in VAMP8KD cells with or without RM treatment. **a** Overlap of autophagosomes and lysosomes with or without RM treatment in GFP-LC3B-expressing HeLa cells with VAMP8KD. **b** Close-up views of lysosome-autophagosome association for the enlarged region indicated by white rectangles in **a**. **c** Percentile distribution of lysosomes unassociated, in contact, and fused with autophagosomes. Data in **c** are presented as mean ± SEM, n.s. refers no significance.


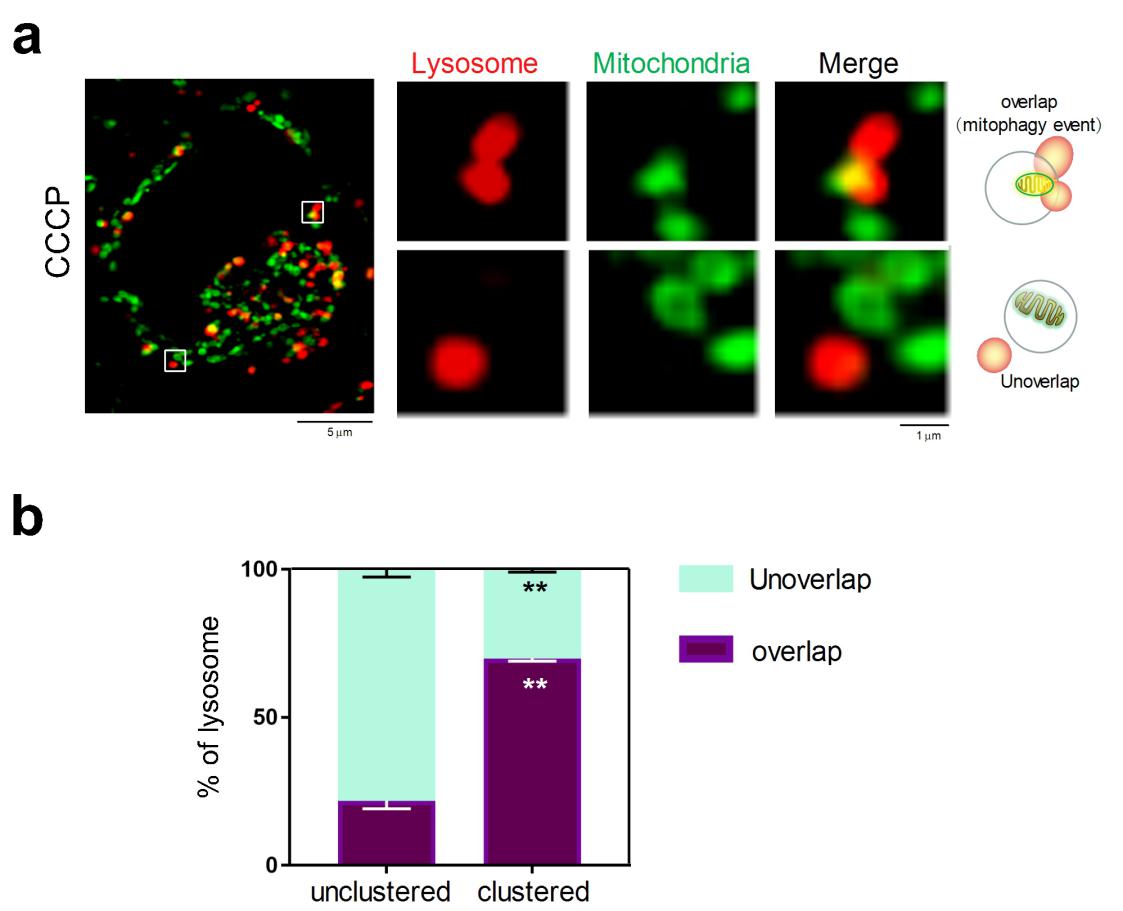


**Supplementary Figure S7** Clustered lysosomes showing a high probability of overlap with damaged mitochondria. **a** Imaging of lysosome–mitochondrion associations in CCCP-treated HeLa cells using SIM. White rectangles indicate representative clustered or unclustered lysosomes. **b** Percentage of unclustered and clustered lysosomes overlap or unoverlap with mitochondria. More than 20 cells from five images were used for the analysis using ImageJ. Data in **b** are presented as mean ± SEM, ***p* < 0.001.


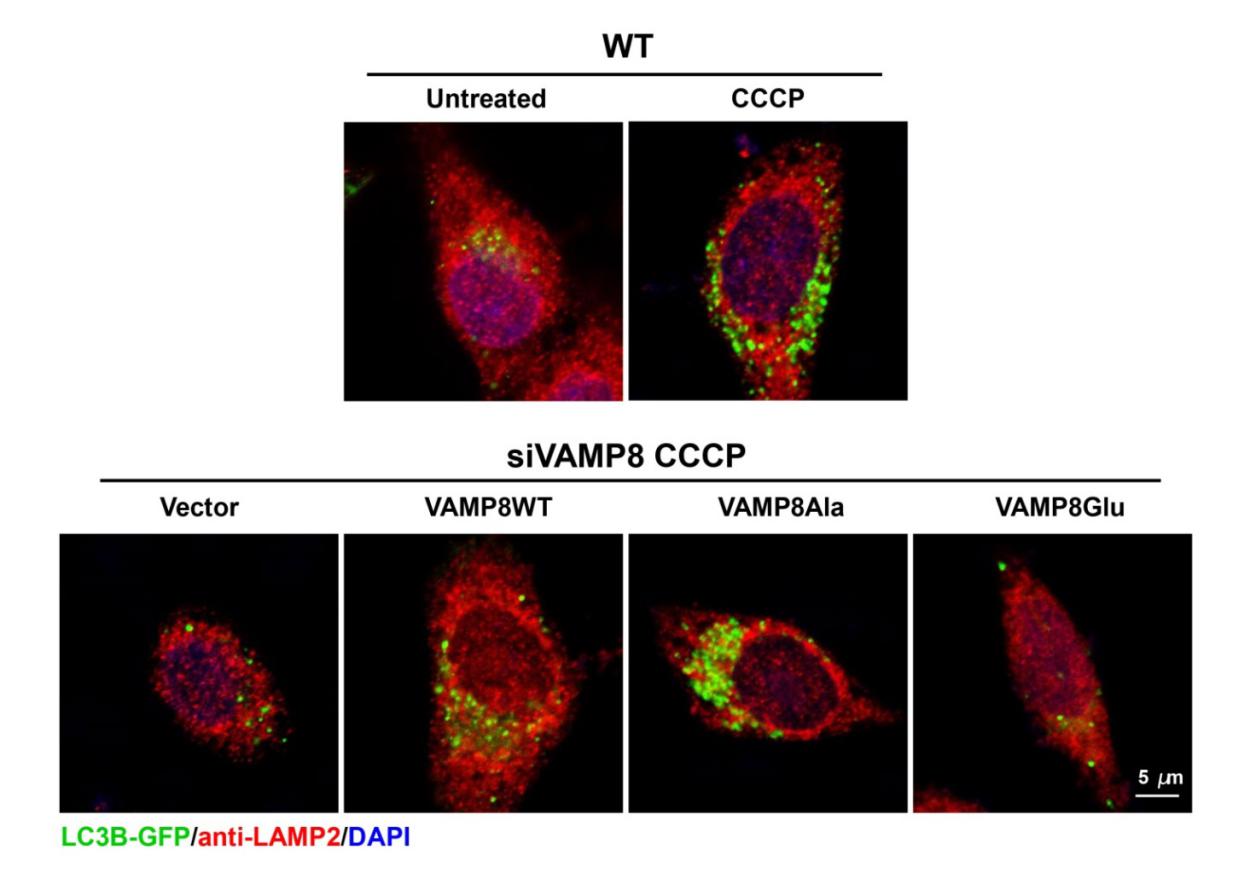


### Supplementary Figure S8 Confirmation of autophagy flux with LC3B. Overlap of LC3B-GFP (green) and LAMP2 (red) in untreated and CCCP-treated WT Hela cells as well as CCCP-treated and siVAMP-8-transfected VAMP8KD, WT rescue, VAMP8Ala, and VAMP8Glu cells. Scale bar: 5 μm.


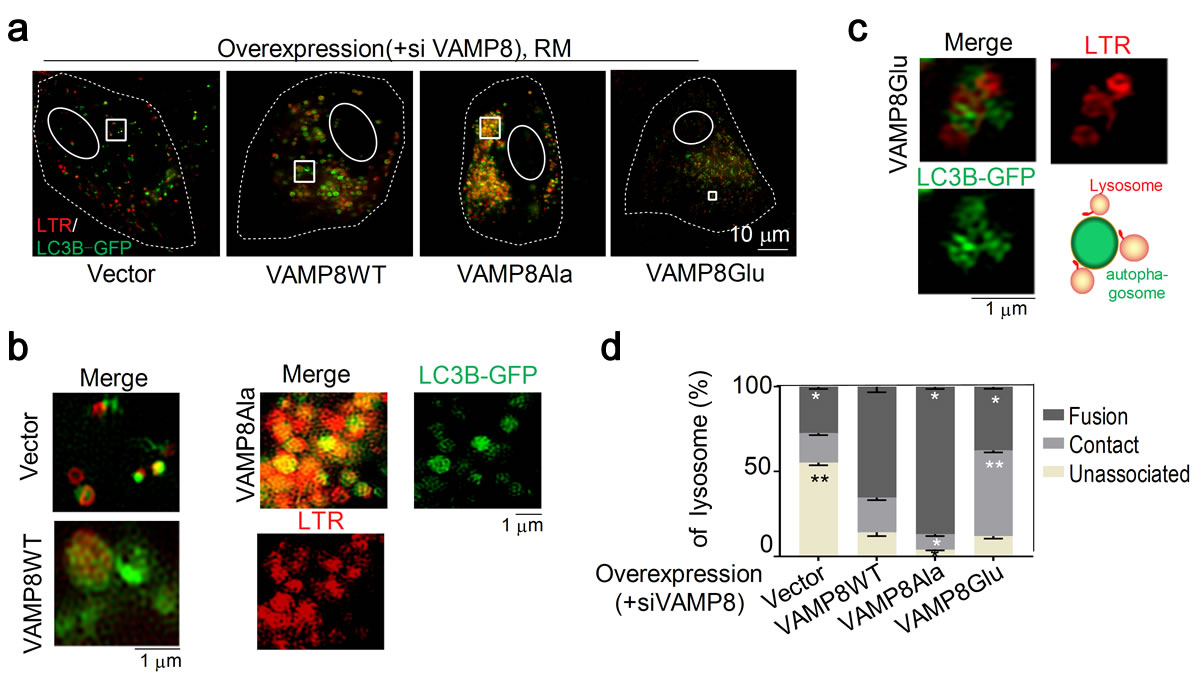


**Supplementary Figure S9** SIM imaging of RM-treated HeLa cells stably expressing GFP-LC3B. **a** Overlap of autophagosomes and lysosomes in RM-treated VAMP8KD, WT rescue, VAMP8Ala mutants (i.e., T48A, T54A, S55A), and VAMP8Glu mutants cells. HeLa cells stably expressing GFP-LC3B were transfected with or without siVAMP8 and mutant vector for 48 h, lysosomes was stained with 200 nM LTR for 30 min to allow the investigation of lysosome–autophagosome association using SIM. **b** Close-up views of SIM images of fusion in vector, VAMP8WT, VAMP8Ala mutants cells for the enlarged region indicated by white rectangles in **a**. **c** Close-up views of SIM images of lysosomes in association with autophagosomes in VAMP8Glu mutants cells. **d** Percentile distribution of lysosomes unassociated, in contact, and fused with autophagosomes in RM-treated VAMP8KD, WT rescue, VAMP8Ala mutants, and VAMP8Glu mutants HeLa cells. Data in **d** are presented as mean ± SEM, **p* < 0.05, ***p* < 0.01.


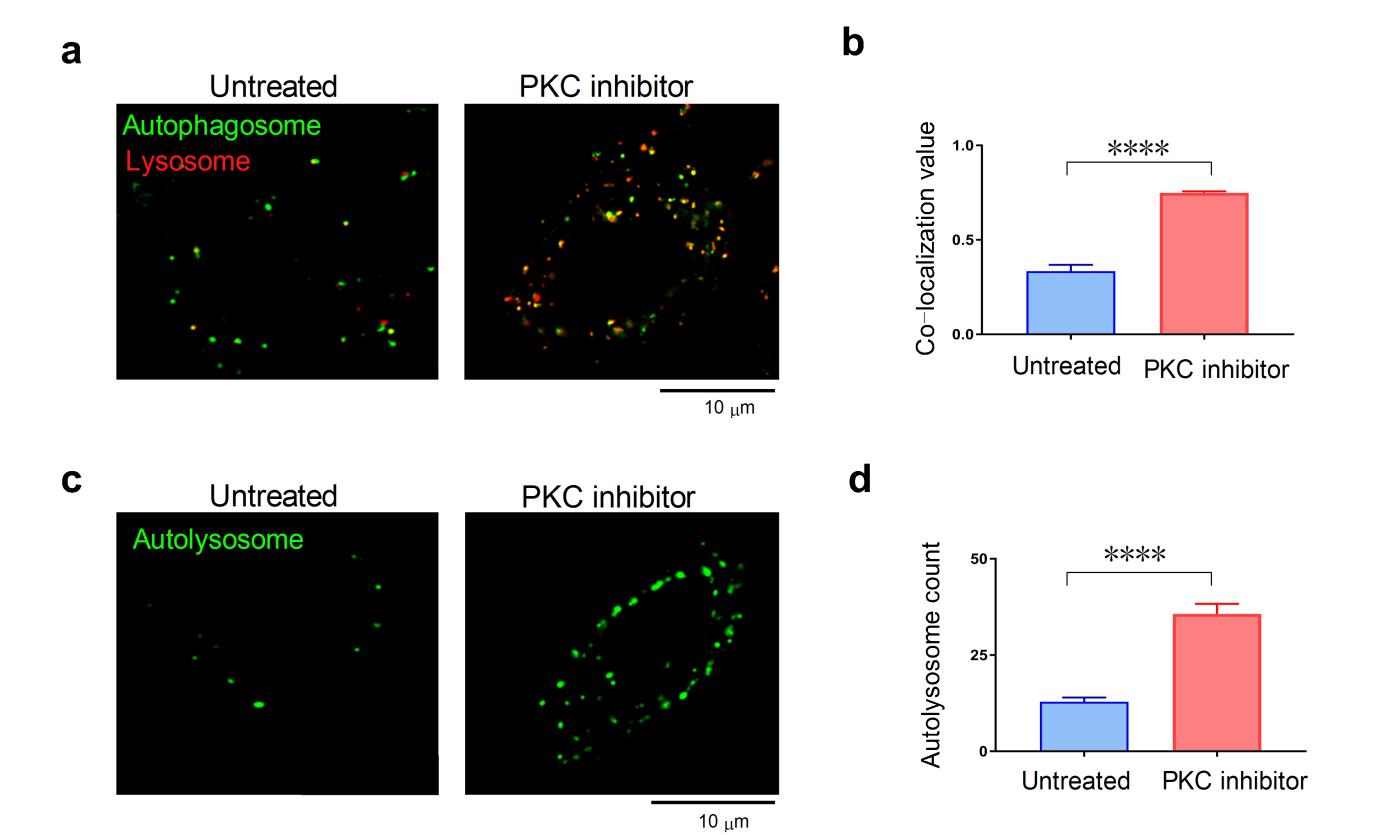


**Supplementary Figure S10** Increased autophagosome–lysosome fusion with PKC inhibitor. **a** Joint localization of lysosomes and autophagosomes with or without 3 μM of PKC inhibitor (Go 6976) treatment for 1 h and **b** the joint localization value. **c** Autolysosome formation with or without 3 μM of PKC inhibitor (Go 6976) treatment and **d** its count. Data in (**b**) and (**d**) are presented as mean ± SEM, *****p* < 0.0001.
